# Tolerogenic Splenic IDO^**+**^ Dendritic Cells from the Mice Treated with Induced-Treg Cells Suppress Collagen-Induced Arthritis

**DOI:** 10.1155/2014/831054

**Published:** 2014-10-27

**Authors:** Jie Yang, Yiming Yang, Huahua Fan, Hejian Zou

**Affiliations:** ^1^Division of Rheumatology, Huashan Hospital, Fudan University, Shanghai 200040, China; ^2^Blood Engineering Laboratory, Shanghai Blood Center, Shanghai 200051, China

## Abstract

TGF-*β*-induced regulatory T cells (iTregs) retain Foxp3 expression and immune-suppressive activity in collagen-induced arthritis (CIA). However, the mechanisms whereby transferred iTregs suppress immune responses, particularly the interplay between iTregs and dendritic cells (DCs)* in vivo*, remain incompletely understood. In this study, we found that after treatment with iTregs, splenic CD11c^+^DCs, termed “DC_iTreg_,” expressed tolerogenic phenotypes, secreted high levels of IL-10, TGF-*β*, and IDO, and showed potent immunosuppressive activity* in vitro*. After reinfusion with DC_iTreg_, marked antiarthritic activity improved clinical scores and histological end-points were observed. The serological levels of inflammatory cytokines and anti-CII antibodies were low and TGF-*β* production was high in the DC_iTreg_-treated group. DC_iTreg_ also induced new iTregs* in vivo*. Moreover, the inhibitory activity of DC_iTreg_ on CIA was lost following pretreatment with the inhibitor of indoleamine 2,3-dioxygenase (IDO). Collectively, these findings suggest that transferred iTregs could induce tolerogenic characteristics in splenic DCs and these cells could effectively dampen CIA in an IDO-dependent manner. Thus, the potential therapeutic effects of iTregs in CIA are likely maintained through the generation of tolerogenic DCs* in vivo*.

## 1. Introduction

Regulatory T cells (Tregs) play an essential role in immune regulation and autoimmune disease prevention. Recently, Tregs have been considered as promising candidates in cell therapy for autoimmune diseases, including rheumatoid arthritis (RA), systemic lupus erythematosus (SLE), and experimental autoimmune encephalitis (EAE) [1]. In a previous study, we showed that transforming growth factor-beta- (TGF-*β*-) induced CD4^+^Foxp3^+^Tregs (iTregs), not natural Tregs (nTregs), could retain immune-suppressive activity, thereby inhibiting bone erosions and clinical measures of disease progression in collagen-induced arthritis (CIA) [2]. However, iTregs are short-lived cells [3], and dendritic cells (DCs) and continuous antigen stimulation are indispensable for the persistent antiarthritis effect of iTregs.

DCs are potent antigen-presenting cells (APCs) regarded as the master regulators of host immunity. The DC populations in specific lymphoid tissue possess distinct characteristics, reflecting disparities in maturation states and immunological environments [4]. “Tolerogenic DC” (tDC) is a blanket description for alternatively activated or specific subset of immature, immune-regulatory DCs [5]. tDCs suppress autoimmunity through various mechanisms, such as modulating inflammatory cytokine secretion and polarizing the Th17/Treg balance [6] via indoleamine 2,3-dioxygenase- (IDO-) dependent mechanisms [7].

In 1993, Qin et al. first described the capacity of CD4^+^T cells to confer suppressive activity to other T cells, a phenomenon termed “infectious tolerance” [8]. Subsequent studies have shown that Tregs induce conventional T cells to become additional tolerant Foxp3^+^ cells through this mechanism, which requires the interplay between Tregs and DCs through TGF-*β* in chronic graft-versus-host disease (cGVHD) mice [9]. In addition, IDO, a catabolic enzyme responsible for the degradation of the essential amino acid tryptophan via the kynurenine pathway, also plays an important role in the crosstalk between Tregs and DCs [10]. And as H. Waldmann et al. reported, further upregulation of not only IDO, but also at least 4 other EAA-consuming enzymes could act to limit T cell proliferation and induce new Tregs via infectious tolerance [11]. However, until recently, the mechanisms of iTreg-induced “infectious tolerance” have remained elusive.

To understand the mechanisms by which iTregs suppress immune responses, particularly the crosstalk between these cells and DCs, we examined the characteristics of DC subsets in the spleens of iTreg-treated CIA mice. We focused on whether these splenic DCs could obtain suppressive activity and promote the generation of new iTregs generation, thereby regulating immunity and maintaining oral tolerance. In the present study, we observed that splenic DCs, particularly IDO-producing CD11b^+^DCs, exhibited tolerogenic characteristics and potential inhibitory activity against CIA following the adoptive transfer of iTregs.

## 2. Materials and Methods

### 2.1. Mice

Eight-week-old wild-type male DBA/1J (D1) mice were obtained from the Shanghai Laboratory Animal Center of the Chinese Academy of Science (SLACCAS, China). All mice were housed in a pathogen-free environment and were treated according to the guidelines of the Institutional Animal Care and Use Committee of the Chinese Association for Laboratory Animal Sciences.

### 2.2. Ethics Statement

This study was carried out in strict accordance with the recommendations in the guidelines of the Institutional Animal Care and Use Committee of the Chinese Association for Laboratory Animal Sciences. The protocol was approved by the Committee on the Ethics of Animal Experiments of Shanghai Blood Center. All surgeries were performed under diethyl ether, and all efforts were made to minimize suffering.

### 2.3. Induction and Evaluation of CIA

CIA was induced in D1 mice after immunizing twice on Day 0 and Day 21 with bovine type II collagen (CII, Chondrex, Redmond, WA, USA) [6]. After 25 days, the onset of CIA was confirmed. At 3 weeks after immunization, the occurrence of limb joint arthritis in the mice was scored every two days using the following established scoring system: no detectable arthritis, 0; erythema and mild swelling, 1; mild erythema and mild swelling involving the entire paw, 2; severe swelling and redness from the ankle to digits, 3; and maximal swelling and redness or obvious joint destruction associated with visible joint deformity or ankylosis, 4. The clinical scores for each mouse are presented as the sum of the scores for the four limbs, and the maximal score for each mouse was sixteen. During the 49-day-observation period, the Arthritis scores rose quickly and constantly and CIA developed in up to 90% of mice.

### 2.4. *Ex Vivo* Generation of CD4^+^iTregs

As previously described [12], CD4^+^CD25^−^T cells were purified from the spleens of D1 mice using a CD4^+^CD25^+^ regulatory T Cell Isolation Kit (Miltenyi Biotec, Bergisch Gladbach, Germany) and were subsequently stimulated with anti-CD3/CD28 monoclonal antibodies (mAbs, 50 ng/mL, PeproTech) in the presence of TGF-*β*1 (5 ng/mL, PeproTech) for 5 days. The T cells were incubated with anti-mouse CD4-FITC mAb, fixed, permeabilized, and stained with anti-mouse FoxP3-PE or isotype control mAbs (BD Biosciences Pharmingen). And the suppressive activity of these cells against T cell proliferation was examined using an* in vitro* suppressive assay. Approximately 3 × 10^6^ cells were transferred to CIA mice at 25 days after the first CII immunization.

### 2.5. Isolation and Characteristic Profile of Splenic DCs

Single mononuclear cells were prepared from the spleens of CIA and iTreg-treated mice and were subsequently isolated using a Mouse DC Enrichment Kit (Dynal, Invitrogen). The purity of the sorted CD11c^+^DCs was >95%. The DCs were stained with mAbs against CD11c, CD11b, CD8*α*, CD80, CD86, CD40, and IA/IE (BD Biosciences Pharmingen). The cells were further fixed, permeabilized, and stained with a rabbit anti-IDO polyclonal antibody (Biolegend) and subsequently analyzed through flow cytometry [13]. The cytokines and function-associated molecules from isolated DCs were quantified using real-time quantitative PCR [5].

For the proliferation assay, responder CD4^+^/CD8^+^T cells were isolated from the spleens of CIA mice (at 25 days after primary immunization) using a CD4^+^/CD8^+^ T Cell Isolation Kit (Miltenyi Biotec, Germany) and were labeled with carboxyfluorescein succinimidyl ester (CFSE; Invitrogen, Germany). The isolated DCs (as suppressor cells) were loaded with CII (20 ng/mL) and subsequently added to the responder cultures, in which the ratio of S : R was 1 : 5. The frequency of dividing CFSE^+^ cells in the coculture was analyzed after 4 days via flow cytometry.

For the suppression assay, responder CD4^+^/CD8^+^ T cells were stimulated with anti-CD3/28 mAbs (50 ng/mL, PeproTech) and cocultured with CII-loaded DCs. After 4 days, the suppressive activity was measured as the frequency of dividing CFSE^+^ cells in the presence or absence of different types of isolated DCs.

### 2.6. Evaluation of CIA after the Reinfusion of Isolated Splenic DCs

Upon the onset of CIA, approximately 5 × 10^5^ isolated splenic DCs were pulsed with CII peptide and adoptively transferred via the tail vein into CIA recipient mice (*n* = 5 per group). Control mice were treated with PBS alone. The mice were scored using an established scoring system, and the mice were sacrificed 49 days after the primary immunization. The hind paws of DC-treated and CIA mice were collected and stained with hematoxylin and eosin (H&E). The paw sections were examined using an established scoring system. In addition, the levels of tumor necrosis factor (TNF), interleukin-17 (IL-17), and IL-6 in the serum were determined using a mouse CBA Kit (BD Pharmingen). The TGF-*β* concentration was assessed using a mouse TGF-*β* Platinum ELISA kit (eBioscience), and anti-CII antibody production was measured using a standard sandwich ELISA (Chondrex, Redmond, WA, USA) according to the manufacturer's instructions. Five samples were analyzed in each group [6].

### 2.7. IDO Blocking Assay

To block IDO, 1-methyl-tryptophan (1-MT, 500 *µ*M, Sigma, USA) was added to the isolated DC culture and washed after 24 h. The capacity of 1-MT block IDO was evaluated using RT-PCR and Western blot analyses [14]. The suppression of isolated DCs* in vitro* and in CIA mice was compared in the presence or absence of 1-MT.

### 2.8. The Conversion of CD4^+^CD25^−^T Cells into Foxp3^+^Tregs through Coculture with Isolated Splenic DCs

CD4^+^CD25^−^T cells stimulated with mDCs were cocultured* in vitro* with isolated splenic DCs for 4 days, and the incidence of FoxP3 was determined through fluorescence-activated cell sorting (FACS) analysis. To assess the regulatory activity, CD4^+^CD25^+^ cells cocultured with DCs for 4 days or freshly isolated CD4^+^CD25^+^T cells from CIA mice were purified according to CD25 expression, and the capacity of these cells to inhibit the proliferation of activated CD4 responder cells was determined.

### 2.9. Statistical Analysis

The results were analyzed using GraphPad Prism 5.0 software (GraphPad Software, San Diego, CA, USA), and the data are expressed as the means ± SEM. Student's* t*-test was used to assess the statistical significance between two paired groups, and an alpha value of *P* < 0.05 was considered statistically significant.

## 3. Results

### 3.1. The Adoptive Transfer of iTregs Generated* Ex Vivo* via TGF-*β* Suppressed the Development of Established Collagen-Induced Arthritis

**Figure 1:**
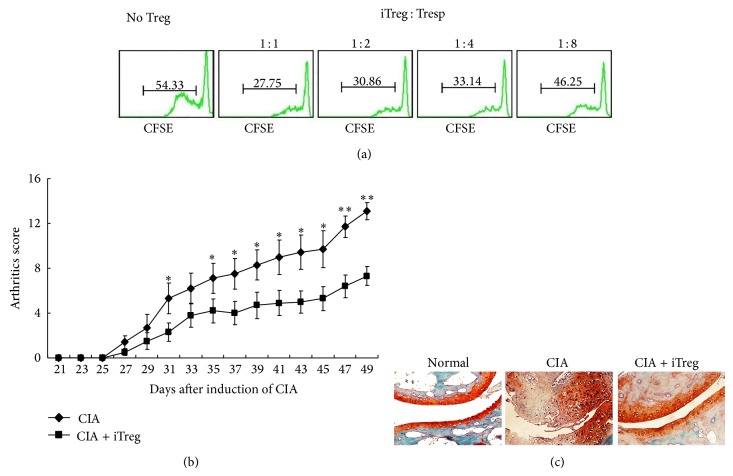
The adoptive transfer of iTregs suppressed the development of CIA. (a) The capacity of iTregs to inhibit CD4^+^T cell proliferation was assessed through CFSE staining. Different doses of iTregs were added to the proliferation assay, and the progressive dilution of CFSE in responder cells was used as a readout of proliferation. (b) CIA mice subjected to the adoptive transfer of iTregs or not were scored for clinical signs of arthritis in the limb joints through macroscopic examination three times a week and were sacrificed 49 days after the first immunization with CII. The arthritic scores in each group (*n* = 10) were expressed as the means ± SEM. ^*^ *P* < 0.05, ^**^ *P* < 0.01 compared with CIA mice using unpaired* t*-tests. (c) Hind paw specimens from recipient mice treated with or without iTregs were collected 3 weeks after the onset of CIA and were stained with Safranin O (showing cartilage erosion).

As previously reported [12], TGF-*β* induced the generation of iTregs from naïve CD4^+^CD25^−^T cells. At 5 days after induction, a large proportion of CD4^+^CD25^−^T cells expressing CD25 and Foxp3 (64.89 ± 6.18%), an important transcription factor for the regulation of the development and function of Tregs, were observed. In addition, the proliferation assay was used as a functional readout to demonstrate the capacity of these iTregs to suppress the proliferation of effector T cells. As shown in Figure 1(a), at all responder-to-iTreg ratios, iTregs suppressed the proliferation of CIA-CD4^+^T cells. Moreover, following the adoptive transfer of 3 × 10^6^ iTregs, marked antiarthritic activity and improved clinical scores (Figure 1(b)) and histological end-points (Figure 1(c)) were observed. These results suggest that iTregs were therapeutically beneficial in CIA.

### 3.2. The Adoptive Transfer of iTregs Induced Tolerogenic Characteristics in Splenic DCs from CIA Mice

Previous studies have shown that transferred iTregs have a limited lifespan in the recipient mice [3], but the long-term (at least 25 days) protective effects of these cells for the prevention of CIA development were demonstrated in the present study. As the induction of tolerance and organ-specific autoimmunity* in vivo* indicated a close relationship with bidirectional crosstalk between DCs and Tregs [15], we tested the characteristic profile of splenic DCs obtained from iTreg-treated CIA mice, CIA mice, and wild-type controls (DC_iTreg_, DC_CIA_, and DC_WT_, resp.).

**Figure 2:**
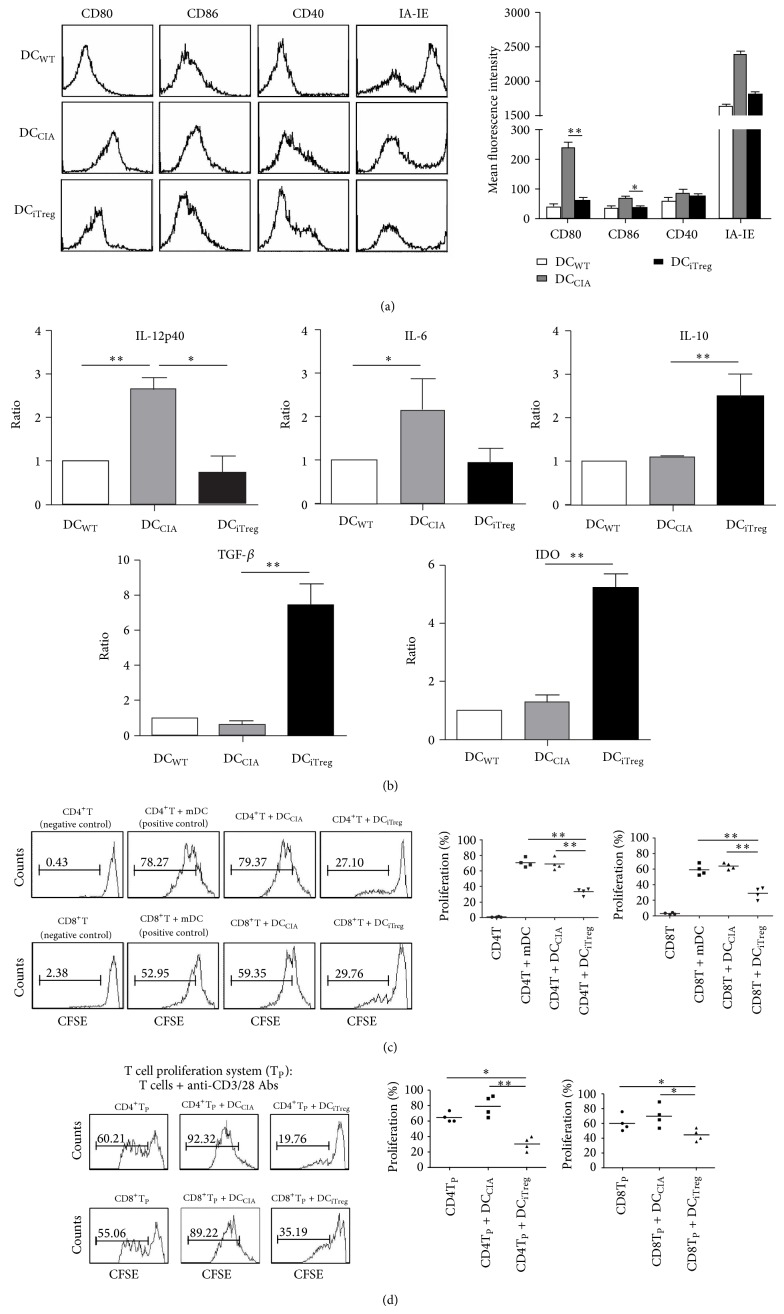
Transferred iTregs induced the tolerogenic characteristics of splenic DCs from CIA mice. (a) After iTreg transfusion, the phenotype of splenic DCs, including CD80, D86, CD40, and IA-IE molecules, was analyzed via flow cytometry. The expressions of the same molecules on DC_WT_ were examined as the isotype control. The data for the mean fluorescence intensities are reported as the means ± SEM (*n* = 10). ^*^ *P* < 0.05, ^**^ *P* < 0.01 compared with the indicated groups using unpaired* t*-tests. (b) IL-12p40, IL-6, IL-10, TGF-*β*, and IDO expression levels in splenic DCs were determined using real-time PCR. All results were normalized to the expression of the housekeeping gene *β*-actin and are expressed as the means ± SEM of six independent experiments. ^*^ *P* < 0.05, ^**^ *P* < 0.01 compared with the indicated groups using unpaired* t*-tests. (c) The capacity of DCs to promote CD4^+^/CD8^+^T cell proliferation was assessed through CFSE staining. Conventional mDCs (as positive controls), DC_CIA_, or DC_iTreg_ were pulsed with CII peptide and added to the CD4^+^/CD8^+^T cell culture assay. The progressive dilution of CFSE in responder cells was used as a readout of proliferation. ^**^ *P* < 0.01 compared with the indicated groups using unpaired* t*-tests. (d) In the proliferation system (T_P_), CD4^+^T/CD8^+^T cells (responders) were isolated from CIA mice SC, stained with CFSE, and stimulated with anti-CD3/28 mAbs. DC_CIA_ or DC_iTreg_ were pulsed with CII peptide and added to the culture at a suppressor : responder (S : R) ratio of 1 : 5. After coculture for 4 days, the cells were harvested and analyzed via FACS analysis. The progressive dilution of CFSE was used as a measure of proliferation. The results are representative of four independent experiments. ^*^ *P* < 0.05 and  ^**^ *P* < 0.01, compared with the DC_iTreg_ group using unpaired* t*-tests.

At 7 days after the adoptive transfer of iTregs into CIA mice, CD11c^+^DCs were isolated from the spleens of different mouse groups, with purities as high as 95%. Compared with DC_WT_, DC_CIA_ expressed obviously higher levels of major histocompatibility complex (MHC) (IA-IE) and costimulatory (CD80, CD86, and CD40) molecules. However, after treatment with iTregs, the mean fluorescence intensity (MFI) of these molecules was consistently lower in DC_iTreg_ than in DC_CIA_, particularly CD80/86 (Figure 2(a)), though the expression of these molecules could not recover to the status of them in the wild-type mice. This result suggests that the adoptive transfer of iTregs could suppress the upregulation of MHC and the expression of costimulatory molecules in splenic DCs, retaining the tolerogenic surface phenotype of these cells.

Cytokine production and the expression of function-associated molecules in DCs were also analyzed using real-time PCR. The results showed negligible IL-12p40 and IL-6 expression in DC_iTreg_, while IL-10 and TGF-*β* were expressed at high levels. However, DC_CIA_ showed the opposite expression profiles of these cytokines. Specifically, significantly enhanced levels of IDO were detected in DC_iTreg_ (Figure 2(b)). These results suggest that the adoptive transfer of iTregs could reduce the expression of inflammatory cytokines and, more importantly, could induce the expression of “immunosuppressive” cytokines and IDO in splenic DCs* in vivo*.

As potent APCs, conventional DCs, particularly mature DCs, activate effector T cells during inflammation [4]. Therefore, the capacity of splenic DCs to expand effector CD4/CD8 T cells was assessed. As shown in Figure 2(c), only approximately 30% of CD4 or CD8 T cells were expanded by DC_iTreg_. However, after coculture with DC_CIA_, both CD4 and CD8 T cells exhibited vigorous proliferation, generating large numbers of dividing T cells, with nearly 80% and 60% expansion, respectively. Thus, after the transfer of iTregs, the capacity of DCs to expand effector T cells was poor.

The T cell proliferation assay was used as a functional readout to demonstrate the capacity of DCs to suppress the proliferation of effector CD4/CD8 T cells. In this assay, CD4/CD8 T cells (responder cells) were isolated from CIA mice and stimulated using anti-CD3/28 mAbs. In the absence of suppressors, the responder cells underwent vigorous proliferation. However, this intense proliferation was significantly suppressed in the presence of DC_iTreg_ (suppressors), cultured at a 1 : 5 suppressor-to-responder cell ratio. In contrast, the addition of DC_CIA_ promoted the proliferation of the responder cells (Figure 2(d)). These data indicated that after iTreg treatment, splenic DCs from CIA mice exhibited a highly effective inhibitory potency.

Taken together, the results suggest that after the adoptive transfer of iTregs, splenic DCs from CIA mice exhibited a series of tolerogenic characteristics, including a tolerogenic surface phenotype, increased secretion of high levels of “immunosuppressive” cytokines and IDO, poor capacity to expand effector T cells, and effective inhibitory potency.

### 3.3. The Adoptive Transfer of iTregs Induced a Significant Inhibitory Effect of Splenic DCs on CIA

As the results obtained in the present study suggest that DC_iTreg_ exhibit significant inhibition* in vitro*, we reinfused additional CIA mice with 5 × 10^5^  DC_iTreg_ and further evaluated the* in vivo* efficacy of these cells.

**Figure 3:**
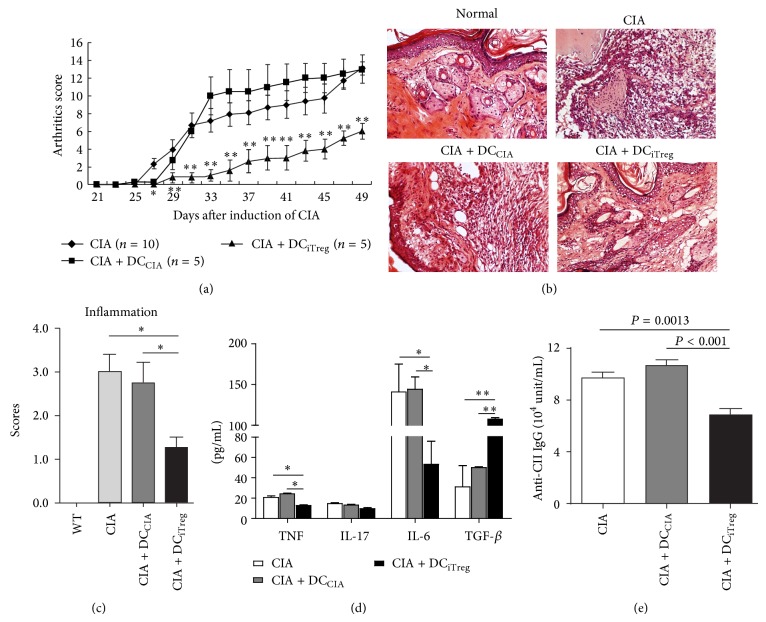
The adoptive transfer of iTregs induced a significant inhibitory effect of splenic DCs on CIA. DC_CIA_ and DC_iTreg_ were pulsed with CII peptide and adoptively transferred into CIA mice at the same density (5 × 10^5^/animal). (a) Each group of mice (*n* = 10) was scored for clinical signs of arthritis. The arthritic scores following the adoptive transfer of different DCs are shown. ^*^ *P* < 0.05, ^**^ *P* < 0.01 compared with DC_iTreg_-treated group and DC_CIA_-treated group/untreated CIA group. (b) Hind paws were collected 3 weeks after the onset of CIA. The tissue was stained with H&E to visualize synovial joint inflammation. (c) The histopathological scores of inflammation were expressed as the means ± SEM of five independent experiments. ^*^ *P* < 0.05 compared with the indicated groups using unpaired* t*-tests. (d) Serum was collected from each group of mice 3 weeks after the onset of CIA. Serum levels of TNF-*α*, IL-17, IL-6, and TGF-*β* were measured using CBA and ELISA assays. The data are reported as the means ± SEM. ^*^ *P* < 0.05, ^**^ *P* < 0.01 compared with the indicated groups using unpaired* t*-tests. The results of five replicated independent experiments were pooled. (e) Serum levels of total CII-specific immunoglobulin in each group of mice were determined using ELISA. The results of five replicated independent experiments were pooled.

Based on the arthritic scores, DC_iTreg_ markedly decreased the incidence of CIA and the severity of arthritis compared with the untreated CIA control group. More importantly, the injection of DC_iTreg_ inhibited the progression of arthritis during the first 10 days after treatment; thereafter, the severity never reached the level observed in the CIA controls (*P* < 0.01). However, the symptoms in CIA mice treated with the same dose of DC_CIA_ were comparatively accelerated and worse than those in CIA controls, although DC_CIA_ could transiently inhibit CIA during the early phase of established CIA (Figure 3(a)). In addition, histopathological examination revealed that the joints of DC_iTreg_-treated mice showed the least inflammatory cell infiltration compared with CIA control mice, whereas DC_CIA_ treatment did not reduce joint inflammation (Figure 3(b)). These results were consistent with the clinical inflammation scores (Figure 3(c)), suggesting that reinfused DC_iTreg_ potently inhibit the progression of CIA.

To further assess the* in vivo* effects of DC_iTreg_ treatment on CIA, the cytokine secretion profiles in the serum of CIA mice treated with or without DC_iTreg_ and DC_CIA_ were analyzed at 25 days after reinfusion. The levels of TNF and IL-6 were significantly lower and the production of TGF-*β* was higher in CIA mice reinfused with DC_iTreg_ compared with those treated with or without DC_CIA_ (Figure 3(d)). Moreover, the serum levels of anti-CII-specific immunoglobulin (anti-CII IgG), an important antibody for the development of CIA pathology, were also assessed. At 25 days after reinfusion with DC_iTreg_, the secretion of anti-CII IgG was significantly lower than that in CIA control mice. Unfortunately, treatment with DC_CIA_ did not reduce the production of anti-CII IgG (Figure 3(e)). These results suggest that the attenuation of CIA in mice following treatment with DC_iTreg_ reflects the immune-modulating effects on the secretion of various cytokines and anti-CII IgG.

Taken together, these results demonstrated that reinfused DC_iTreg_ reduces the severity of CIA, an effect that is associated with modulated cytokine secretion and the inhibition of anti-CII-specific IgG secretion.

### 3.4. The Adoptive Transfer of iTregs Promoted IDO Secretion from Splenic DCs, Resulting in Further Suppression of CIA

Studies indicating that IDO deficiency accelerates CIA [16] suggest that IDO plays an important immunosuppressive role in arthritis and the crosstalk between Tregs and DCs [10]. As the adoptive transfer of iTregs promotes the expression of high levels of IDO in splenic DCs, we assessed whether these IDO-positive splenic DCs inhibit CIA.

**Figure 4:**
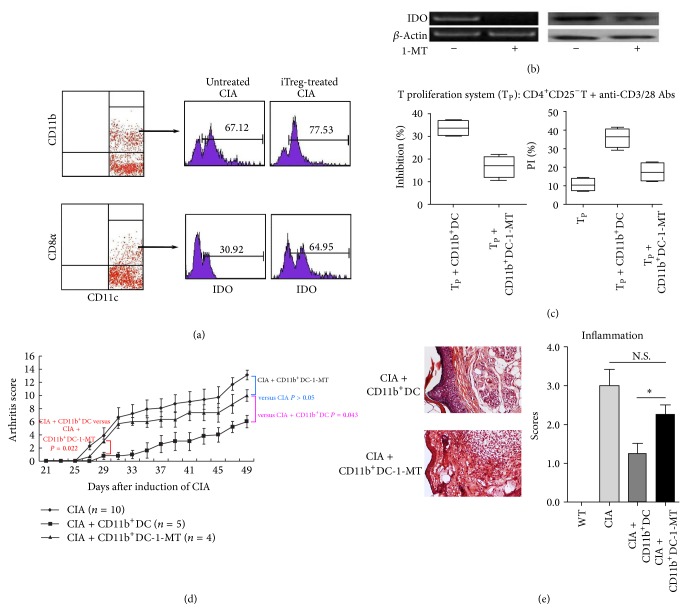
The adoptive transfer of iTregs promoted IDO secretion in splenic DCs and the further suppression of CIA. (a) IDO expression levels in CD11b^+^DCs and CD8*α* ^+^DCs from iTreg-treated CIA mice were determined using FACS. The corresponding DC subsets from untreated CIA mice were used as controls. (b) The results of RT-PCR and Western blotting showed the expression of IDO in CD11b^+^DCs after blocking with 1-MT. (c) CD11b^+^DCs were pretreated with 1-MT to block IDO expression and were subsequently added to the CD4T proliferation system. Four days after cocultivation, CFSE^+^CD4^+^T cells were stained with propidium iodide (PI) and analyzed using flow cytometry. The error bars indicate SEM. The results of 4 replicated experiments were pooled. (d) Recipient mice subjected to the adoptive transfer of CD11b^+^DCs, with or without 1-MT treatment, were sacrificed 49 days after the first CII immunization. The arthritic scores in each group are shown (*n* = 5). (e) Histopathological examination of the joints of mice described in (d) after H&E staining to visualize inflammatory cell infiltration. The histopathological inflammation scores are expressed as the means ± SEM of five independent experiments. ^*^ *P* < 0.05 compared with the indicated groups using unpaired* t*-tests.

First, to identify the characteristics of IDO-expressing DCs in the spleen, the IDO expression levels in CD11b^+^/CD11b^−^ DCs and CD8*α* ^+^/DC8*α* ^−^ DCs from iTreg-treated CIA mice were determined via FACS analysis. After the adoptive transfer of iTregs, IDO expression was higher in all four cell subsets compared with that in the corresponding cell subsets in CIA control mice. Specifically, CD11b^+^ cells showed the highest IDO expression levels among splenic DC subsets at 85%, although nearly 55% of CD8*α* ^+^DCs expressed IDO (Figure 4(a)). These data indicated that CD11b^+^DCs were a major contributor of IDO expression in iTreg-treated CIA mice.

Secondly, to determine the inhibitory effect of IDO^+^DCs from iTreg-treated CIA mice, splenic CD11b^+^DCs were isolated and pulsed with CII* in vitro* with or without 1-MT, a chemical inhibitor of IDO. The results of RT-PCR and Western blotting confirmed that 1-MT effectively inhibited IDO expression in CD11b^+^DCs (Figure 4(b)). As shown in Figure 4(c), CD11b^+^DCs significantly reduced T cell proliferation, and this suppression was reversed after pretreating CD11b^+^DCs with 1-MT, suggesting that IDO was responsible for the suppression of this T cell proliferation. In addition, CD11b^+^DCs promoted the death of responder T cells, and pretreatment with 1-MT reduced the percentage of cell death. These data suggest that CD11b^+^DCs from iTreg-treated mice suppressed CII-induced T cell proliferation and enhanced T cell death through an IDO-dependent mechanism* in vitro*.

Moreover, to confirm whether IDO^+^CD11b^+^DCs from iTreg-treated mice exerted inhibitory effects* in vivo*, approximately 5 × 10^5^ CD11b^+^DCs, pretreated as described above, were reinfused into CIA mice, and the severity of joint inflammation was monitored. Repeated experiments demonstrated remarkable antiarthritis activity and an improved clinical score in CIA mice following the reinfusion of CD11b^+^DCs. As expected, this great suppressive effect was significantly reduced when CD11b^+^DCs were pretreated with 1-MT, indicating that IDO is associated with the induction of immune suppression* in vivo* after vaccination with CD11b^+^DCs (Figure 4(d)). Furthermore, histopathological analyses revealed that the joints of mice treated with CD11b^+^DCs showed a remarkable decrease in inflammatory cell infiltration compared with mice injected with 1-MT-treated DCs (Figure 4(e)). These results were consistent with those obtained from the* in vitro* experiments, suggesting that IDO plays an important role in suppressing arthritis severity.

Taken together, these results further suggest that the adoptive transfer of iTregs promoted IDO secretion from splenic DCs, particularly from the CD11b^+^ subset, and these DCs exhibited potent suppression* in vitro *and in CIA mice through an IDO-dependent mechanism.

### 3.5. The Conversion of Foxp3^+^Tregs from CD4^+^CD25^−^T Cells through Coculture with Isolated Splenic IDO^+^DCs

As the adoptive transfer of iTregs induced splenic tolerogenic IDO^+^DCs, we determined whether these IDO^+^DCs were involved in the generation of CD4^+^Foxp3^+^Tregs* in vitro* or in CIA mice.

**Figure 5:**
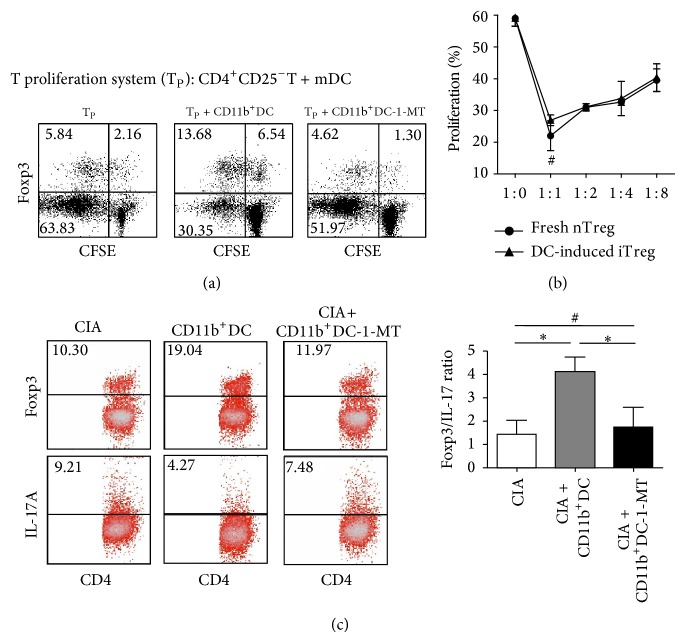
The conversion of Foxp3^+^Tregs from CD4^+^CD25^−^T cells through coculture with isolated splenic CD11b^+^IDO^+^DCs. (a) CD4^+^CD25^−^T cells (10^5^) stimulated with mDCs (2 × 10^4^) were cocultured for 4 days with isolated splenic CD11b^+^DCs (10^4^) with or without 1-MT pretreatment. The cells were surface-stained with anti-CD4 mAbs, followed by intracellular staining with anti-Foxp3 mAbs to determine the frequency of CD4^+^Foxp3^+^Tregs. The dates are presented using a dot plot, expressed as the % positive cells. The results are representative of three experiments showing similar results. (b) After cocultivation for 4 days, as described in (a), CD11b^+^DC-induced CD4^+^CD25^+^T cells were isolated and used as inhibitors at different doses to suppress the proliferation of new CD4^+^CD25^−^T cells in response to stimulation with anti-CD3/28 mAbs. The suppressive activity of freshly isolated CD4^+^CD25^+^T cells was also examined as a control. The proliferative responses were measured after 4 days. The data are representative of three experiments with similar results, reported as the means ± SEM. ^#^ *P* > 0.05 compared with the indicated groups using unpaired* t*-tests. (c) CD4^+^T cells from the spleens of CIA mice treated with CD11b^+^DC with or without 1-MT on the third week after the onset of arthritis were collected and intracellularly stained with anti-Foxp3 and anti-IL-17A mAbs. FACS was used to measure the percent frequency of positively stained cells, and the frequencies of Treg/Th17 cells are expressed as the means ± SEM of four independent experiments. ^#^ *P* > 0.05, ^*^ *P* < 0.05 compared with the indicated groups using unpaired* t*-tests.

To determine the effects of these cells* in vitro*, splenic CD11b^+^DCs isolated from iTreg-treated CIA mice were added to the CD4^+^CD25^−^T cell proliferation system, and after 4 days, the frequency of Foxp3^+^Tregs was analyzed via FACS. As indicated in Figure 5(a), the addition of CD11b^+^DCs markedly impaired Foxp3^−^T cell proliferation. Oppositely, the percentage of Foxp3 in effector cells was increased, suggesting that CD11b^+^DCs promoted the conversion of naïve CD4^+^CD25^−^Foxp3^−^T cells into adoptive CD4^+^Foxp3^+^Tregs. Furthermore, these DCs lost inhibitory activity when pretreated with 1-MT.

Next, considering that the natural frequency of Foxp3^+^ cells in mice is >90% compared with CD4^+^CD25^+^ cells [17], CD4^+^CD25^+^ cells from CD11b^+^DCs cultures induced for 4 days were purified based on CD25 expression, and the inhibitory effects of these cells were then assessed. Similar to CD4^+^CD25^+^T cells freshly isolated from CIA mice, CD11b^+^DC-induced CD4^+^CD25^+^T cells effectively suppressed CD4^+^CD25^−^T cell proliferation, and no significant differences in suppression were observed at Treg : Tresp ratios (1 : 1, 1 : 2, 1 : 4, and 1 : 8) (Figure 5(b)).

Moreover, splenic CD11b^+^IDO^+^DCs isolated from iTreg-treated CIA mice were pulsed with CII and reinfused into new CIA mice. At 25 days following the onset of arthritis, the percent frequencies of Th17 (CD4^+^IL-17^+^) and Treg (CD4^+^Foxp3^+^) cells among CD4^+^T cells isolated from the spleen of CIA mice treated with or without CD11b^+^DCs were determined. As shown in Figure 5(c), an increase in the number of Tregs and a decrease in the percent frequency of Th17 cells were observed following treatment with CD11b^+^DCs, resulting in an increase in the Foxp3 : IL-17 ratio. Furthermore, blockage of DCs through 1-MT treatment did not alter the proportion of Treg/Th17 cell populations.

These results suggest that CD11b^+^IDO^+^DCs induced the generation and proliferation of functional CD4^+^Foxp3^+^Treg cells* in vitro* and in CIA mice.

## 4. Discussion

Foxp3^+^ regulatory T cells play a crucial role in maintaining immune tolerance [18]. Previous studies have demonstrated the potential use of nTregs for the treatment of autoimmune diseases [19]. However, several studies have also indicated the limitations of these cells during inflammation [20, 21]. It has been reported that exogenous polyclonal iTregs retain Foxp3 expression and immune-suppressive activity in mouse models of SLE, diabetes, and RA [2, 22, 23]. However, the mechanisms underlying the iTreg-mediated suppression of immune responses are not completely understood. Although initial experiments showed that TGF-*β* signaling was indispensable for the suppression of the immune response by iTregs [9] and that iTregs directly shifted the balance between Tregs and Th17 cells [2], the aim of the present study was to determine whether other indirect mechanisms were involved in iTreg-based suppression, particularly the crosstalk between iTregs and other cell types, such as DCs.

**Figure 6:**
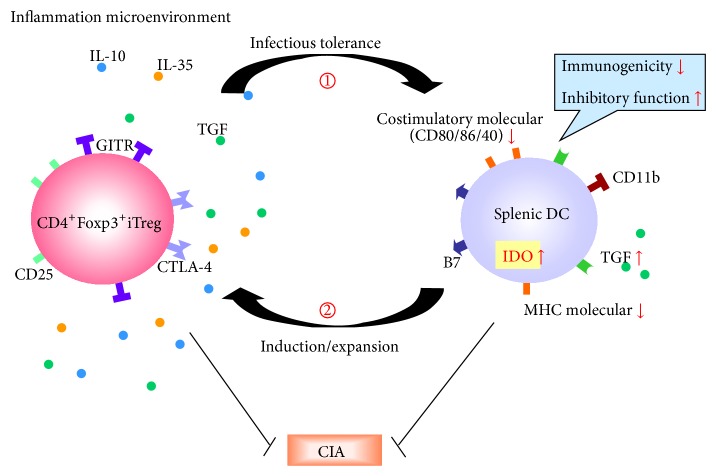
The crosstalk between iTregs and splenic DCs in CIA mice. The feedback loop generated through crosstalk between iTreg and splenic DCs* in vivo*. Transferred iTregs induce a series of tolerogenic characteristics in splenic DCs, including the reduced costimulatory molecule expression, increased TGF-*β* secretion, and reduced immunogenicity and effective inhibitory function. Furthermore, these tolerogenic DCs also exhibited protective effects on CIA. In addition, these DCs convert CD4^+^Foxp3^−^ T cells into new Foxp3^+^iTregs* in vivo*, which inherited the potential suppressive activity and maintained oral tolerance. Obviously, the long-term protection against CIA was influenced through the close relationship and continuous interaction of DCs and iTregs, termed “infectious tolerance.”

In the present study, we provided evidence of therapeutically important interactions between iTregs and splenic DCs in CIA mice. After adoptive transfer, iTregs demonstrated a limited lifespan in recipient mice [3], but the suppressive effects of these cells on the progression of CIA had long-term consequences. The transferred iTregs induced a series of tolerogenic characteristics in splenic DCs, including reduced costimulatory molecule expression, increased TGF-*β* secretion, reduced immunogenicity, and effective inhibitory activity* in vitro*. Furthermore, the infusion of these DCs into new CIA mice showed protective effects equivalent to those of the initially transferred iTregs. Moreover, these DCs could convert CD4^+^Foxp3^−^T cells into additional Foxp3^+^iTregs both* in vitro* and* in vivo*, and these cells demonstrated potential suppression and maintained oral tolerance. Obviously, the long-term protection against CIA was mediated through crosstalk between DCs and iTregs, suggesting that these tolerogenic DCs are key players in Treg-induced “infectious tolerance” (Figure 6). The constant cycling of this feedback loop might stabilize and even enhance the effects of iTreg treatment.

Clearly, during Treg-induced “infectious tolerance,” splenic DCs were shifted into a tolerogenic cell subset, termed splenic tDCs. Interestingly, in a previous study, we successfully propagated another subset of tDCs from bone marrow (BM) cells using exogenous IL-10 and TGF-*β*1* in vitro*. These BM-derived tDCs were comparatively resistant to maturation and were highly effective in the suppression of T cell proliferation* in vitro*, even at low doses. Moreover, in CIA mice, tDCs suppressed the progression of established CIA, associated with the modulation of cytokine secretion, polarization of the Treg/Th17 ratio, and inhibition of anti-CII secretion [6]. Similar results were also observed with splenic tDCs in the present study, suggesting that the adoptive transfer of iTregs induced IL-10 and TGF-*β*1 production during inflammation* in vivo* and retained oral tolerance, thereby promoting splenic tDC induction. Lan et al. also showed that the TGF-*β* signal is crucial for the effects of iTregs on DCs [9]. In addition, we presumed DCs residing other issues, especially joint, were also modified following iTregs infusion, which seemed to exert their immune effect more directly after induction of oral tolerance in CIA mice, and we would tackle this point experimentally in future.

Previously, it was reported that IDO-producing DCs in Peyer's patches (PPs) and plasmacytoid DCs (pDCs) from draining lymph nodes play essential roles in the induction of oral tolerance [24, 25]. The spleen is an important lymphoid organ responsible for inducing Tregs in the periphery; therefore, we focused on the DCs in the spleen in the present study. Splenic DCs are distinct from the DCs in PPs and pDCs, but these cells showed similar IDO-mediated effects in the induction of tolerance. Notably, splenic DCs induce the secretion of interferon-gamma (IFN-*γ*) [26], an IDO inducer [27], in T cells. Thus, IDO-secreting splenic DCs represent likely candidates for Treg-induced “infectious tolerance.”

Notably, CD11b^+^ splenic DCs are the important players in Treg-DC crosstalk. Park et al. reported that CD11b^+^DCs in PPs are crucial for the establishment of oral tolerance. In IDO-knockout mice, the CD11b^+^DC population was significantly reduced, whereas the proportion of other DC subsets remained unaffected [13]. Thus, CD11b^+^DCs might play a major role in IDO production in DCs, consistent with the results of the present study. Herein, we used FACS analysis to examine IDO expression in splenic CD11b^+^/CD11b^−^ DCs and CD8*α* ^+^/DC8*α* ^−^ DCs from iTreg-treated CIA mice, and the results showed that CD11b^+^ cells exhibited the highest IDO expression (85%) among the splenic DC subsets in iTreg-treated CIA mice. Thus, we further examined the immune-regulatory activity of CD11b^+^DC cells through IDO production and suppression during inflammation in CIA. Additionally, Manlapat et al. showed that CD8*α*+B220+CD19+ splenic DCs also secrete functional IDO [28], and the results of the present study indicated that nearly 55% of CD8*α* ^+^DCs in the spleen expressed IDO after iTreg treatment in CIA mice and this subset upregulated IDO levels even more. As the distinct population, CD8*α* ^+^DCs are considered to contribute to the priming, activation, and function of antitumor CD8T cells and the roles of this subset in CIA mice were determined in our further work. Taken together, these results suggest that the adoptive transfer of iTregs induces several subsets of IDO-secreting DCs, thereby promoting the IDO-dependent suppression of inflammation.

IDO, which degrades the essential amino acid tryptophan, is important in host defenses and immunosuppression for the maintenance of tolerance in cancer, transplantation [29], and EAE [30]. This enzyme also influences the crosstalk between Tregs and DCs [10]. IDO demonstrated an immunosuppressive role in arthritis, based on studies showing that a deficiency in IDO accelerated CIA [16]. In the present study, the immune-regulatory activity of IDO-producing CD11b^+^DCs was demonstrated through the administration of the IDO inhibitor 1-MT. The potent suppression of IDO^+^CD11b^+^DCs* in vitro* and in CIA mice disappeared after 1-MT treatment, suggesting that the antiarthritis effects of splenic tDCs, including the modulation of cytokine secretion, inhibition of anti-CII-specific IgG secretion, and the secondary induction of new iTregs, were IDO dependent. These results further suggest that IDO is a key player in the crosstalk between iTregs and DCs.

In conclusion, the results of the present study showed that iTregs inhibit CIA through the formation of splenic tDCs. The onset of “infectious tolerance” in splenic DCs, particularly CD11b^+^DCs, conferred tolerogenic characteristics in these cells. These splenic tDCs could effectively suppress the severity and progression of CIA in an IDO-dependent manner, associated with the modulation of inflammatory cytokine and anti-CII antibody secretion and the induction of new functional iTregs. Thus, the potential therapeutic effects of iTregs on CIA and RA are likely to stabilize, maintain, and even enhance the effects of DCs* in vivo*.
